# Supplementary material for: In Vivo Rapid Investigation of CRISPR-Based Base Editing Components in Escherichia coli (IRI-CCE): A Platform for Evaluating Base Editing Tools and Their Components
Source: Int J Mol Sci. 2022 Jan 20;23(3):1145. doi: 10.3390/ijms23031145 (PMC8834901; doi:10.3390/ijms23031145)
Supplement: Supplementary file 1 [file ijms-23-01145-s001.zip › ijms-1525626-supplementary.pdf]

Supplementary Material 1

In vivo Rapid Investigation of CRISPR-based Base Editing Components in *Escherichia coli* (IRI-CCE): A Platform for Evaluating Base Editing Tools and Its Components

Rahul Mahadev Shelake[1,†,*], Dibyajyoti Pramanik[1,†], Jae-Yean Kim[1,2,*]

[1]Division of Applied Life Science (BK21 Four Program), Plant Molecular Biology and Biotechnology Research Center, Gyeongsang National University, Jinju 52828, Korea

[2]Division of Life Science, Gyeongsang National University, 501 Jinju-daero, Jinju 52828, Korea

[†]These authors contributed equally to this work.

*** Correspondence:**

Jae-Yean Kim: kimjy@gnu.ac.kr (Orcid ID: 0000-0002-1180-6232)

Rahul Mahadev Shelake: rahultnau@gmail.com (Orcid ID: 0000-0003-0691-560X)

	Content	Page
1	Supplementary Figure S1. Promoter sequences from different organisms used in Figure 1.	3
2	Supplementary Figure S2. Evaluation of promoter activities at transcriptional level in *E. coli*.	4
3	Supplementary Figure S3. Promoter sequences used for sgRNA expression in different organisms.	5
4	Supplementary Figure S4. Analysis of two-plasmid and single-plasmid vector systems for evaluation of C-to-T conversion by PmCDA1-mediated cytosine base editor.	6
5	Supplementary Figure S5. C-to-T conversion by PmCDA1-mediated cytosine base editor designed using nickase (nCas9, D10A) and dead (dCas9, D10A+H840A) form of Cas9.	7
6	Supplementary Figure S6. Cytotoxicity assay of nCas9 and cytosine base editors in different *E. coli* strains.	8
7	Supplementary Figure S7. Cytotoxicity of nCas9(D10A) and cytosine base editors.	9
8	Supplementary Figure S8. Features of inactive sgRNAs from Figure 5.	10
9	Supplementary Figure S9. Editing windows for CBEs (PmCDA1, evoCDA1, APOBEC3A) and ABEs (ABE8e, ABE9e) reported in the present study expressed under the promoters of different strengths.	11
10	Supplementary Table S1. Overview of cytotoxicity observed in different combinations of promoters tested for expression of BE components.	12
11	Supplementary Table S2. DNA sequences of CRISPR and BE components.	13
12	Supplementary Table S3. *Escherichia coli* strains used in the present study.	14
13	Supplementary Table S4. Primer sequences used for cloning and sequencing are summarized.	17
14	Supplementary Table S5. Plasmids used in the present work.	18
15	**References**	22

pEc1 (SJM901+RBSTL2)

TTTACAGCTAGCTCAGTCCTAGGTATAATGCTAGCAGATCTAATAATTTTGTTTAACTTTGGGAGGATA

pGlpT (BBa_J72163 GlpT+RBS)

GAAAGTGAAACGTGATTTCATGCGTCATTTTGAACATTTTGTAAATCTTATTTAATAATGTGTGCGGCAATTCACATTTAATTTATGAATGTTTTCTT
AACATCGCGGCAACTCAAGAAACGGCAGGTTCGGATCTTAGCTACTAGAGAAAGAGGAGAAATACTAG

p35S(L)I [CaMV 35S(Long)+AtUBQ10 Intron I +TMV5'U]

GAATTCCAATCCCACAAAAATCTGAGCTTAACAGCACAGTTGCTCCTCTCAGAGCAGAATCGGGTATTCAACACCCTCATATCAACTACTACGTTGTG
TATAACGGTCCACATGCCGGTATATACGATGACTGGGGTTGTACAAAGGCGGCAACAAACGGCGTTCCCGGAGTTGCACACAAGAAATTTGCCACTAT
TACAGAGGCAAGAGCAGCAGCTGACGCGTACACAACAAGTCAGCAAACAGACAGGTTGAACTTCATCCCCAAAGGAGAAGCTCAACTCAAGCCCAAGA
GCTTTGCTAAGGCCCTAACAAGCCCACCAAAGCAAAAAGCCCACTGGCTCACGCTAGGAACCAAAAGGCCCAGCAGTGATCCAGCCCCAAAAGAGATC
TCCTTTGCCCCGGAGATTACAATGGACGATTTCCTCTATCTTTACGATCTAGGAAGGAAGTTCGAAGGTGAAGGTGACGACACTATGTTCACCACTGA
TAATGAGAAGGTTAGCCTCTTCAATTTCAGAAAGAATGCTGACCCACAGATGGTTAGAGAGGCCTACGCAGCAAGTCTCATCAAGACGATCTACCCGA
GTAACAATCTCCAGGAGATCAAATACCTTCCCAAGAAGGTTAAAGATGCAGTCAAAAGATTCAGGACTAATTGCATCAAGAACACAGAGAAAGACATA
TTTCTCAAGATCAGAAGTACTATTCCAGTATGGACGATTCAAGGCTTGCTTCATAAACCAAGGCAAGTAATAGAGATTGGAGTCTCTAAAAAGGTAGT
TCCTACTGAATCTAAGGCCATGCATGGAGTCTAAGATTCAAATCGAGGATCTAACAGAACTCGCCGTCAAGACTGGCGAACAGTTCATACAGAGTCTT
TTACGACTCAATGACAAGAAGAAAATCTTCGTCAACATGGTGGAGCACGACACTCTGGTCTACTCCAAAAATGTCAAAGATACAGTCTCAGAAGATCA
AAGGGCTATTGAGACTTTTCAACAAAGGATAATTTCGGGAAACCTCCTCGGATTCCATTGCCCAGCTATCTGTCACTTCATCGAAAGGACAGTAGAAA
AGGAAGGTGGCTCCTACAAATGCCATCATTGCGATAAAGGAAAGGCTATCATTCAAGATCTCTCTGCCGACAGTGGTCCCAAAGATGGACCCCCACCC
ACGAGGAGCATCGTGGAAAAAGAAGAGGTTCCAACCACGTCTACAAAGCAAGTGGATTGATGTGACATCTCCACTGACGTAAGGGATGACGCACAATC
CCACTATCCTTCGCAAGACCCTTCCTCTATATAAGGAAGTTCATTTCATTTGGAGAGGACACGCTCGAGTATAAGGTAAATTTCTGTGTTCCTTATTC
TCTCAAAATCTTCGATTTTGTTTTCGTTCGATCCCAATTTCGTATATGTTCTTTGGTTTAGATTCTGTTAATCTTAGATCGAAGATGATTTTCTGGGT
TTGATCGTTAGATATCATCTTAATTCTCGATTAGGGTTTCATAGATATCATCCGATTTGTTCAAATAATTTGAGTTTTGTCGAATAATTACTCTTCGA
TTTGTGATTTCTATCTAGATCTGGTGTTAGTTTCTAGTTTGTGCGATCGAATTTGTCGATTAATCTGAGTTTTTCTGATTAACAGGAGCTCATTTTTA
CAACAATTACCAACAACAACAAACAACAAACAACATTACAATTACATTTACAATTATCGATAC

Supplementary Figure S1. Promoter sequences used in Figure 1.

Additional features like ribosome binding sites (RBSs) in bacterial promoters are underlined. The intron region from *AtUbi10* in p35S(L)I is highlighted in violet, and the TMV 5U leader sequence is depicted in blue.

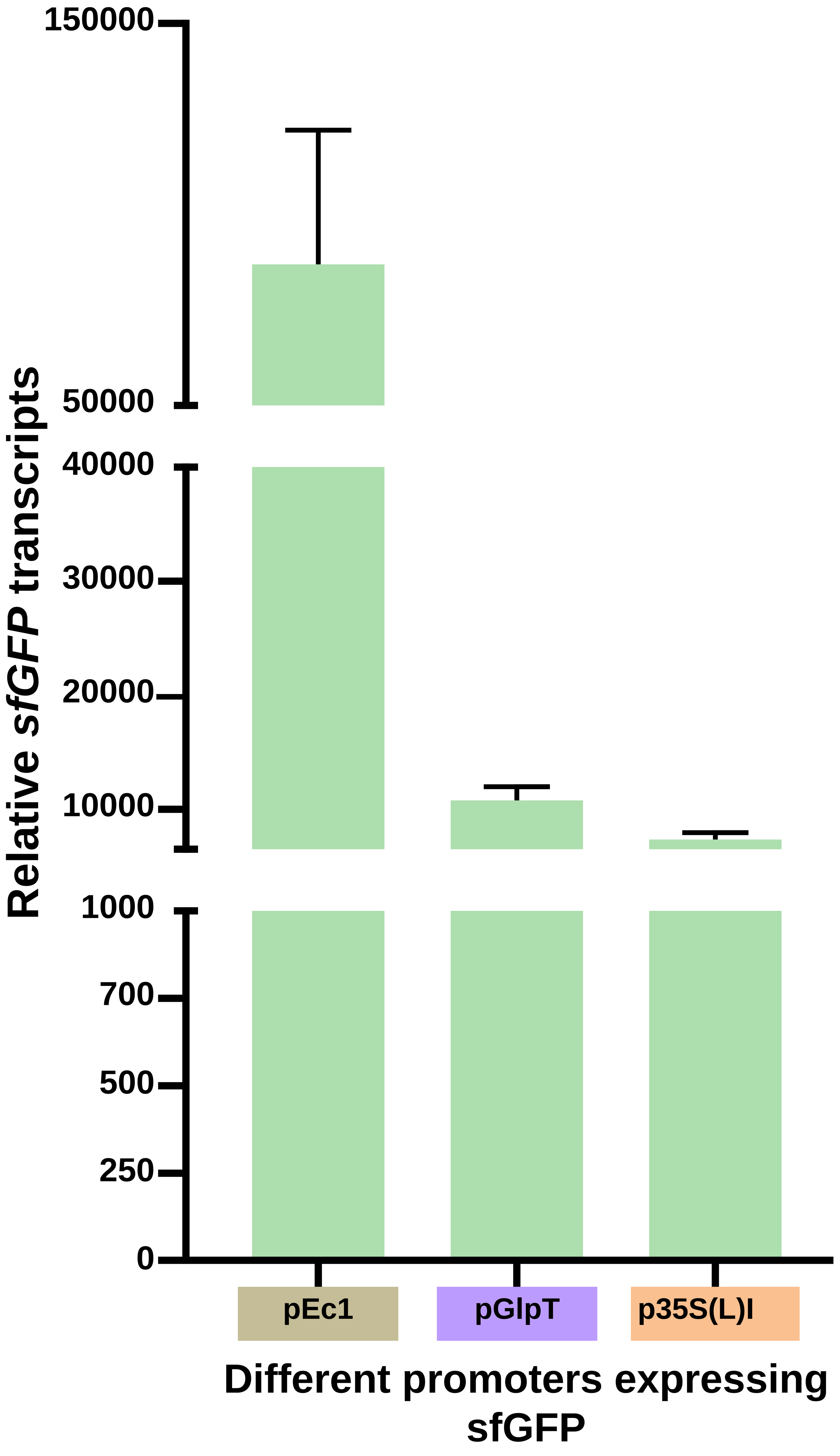

Supplementary Figure S2. Evaluation of promoter activities at transcriptional level in *E. coli*.

Promoters were cloned to drive the *sfGFP* expression. Real-time quantitative PCR was performed to determine the relative *sfGFP* transcript level in *E. coli*. All the values were normalized against internal control 16S ribosomal RNA (*rrsA*) gene Data analyses were conducted using the $^{2-\Delta\Delta C}$t method. Error bars represent standard error (SE). Data represent three biological replicates. Asterisks indicate statistically significant differences (**** p< 0.0001, independent samples t-test).

-10 (TATAAT) element

-35 (TTGACA) element

Bacteria

>pJ23119
TTGACAGCTAGCTCAGTCCTAGGTATAATACTAGT

Plant

>pAtU6
TGATCAAAAGTCCCACATCGATCAGGTGATATATAGCAGCTTAGTTTATATAATGATAGAGTCGACATAGCG

>pOsU3
AAGGAATCTTTAAACATACGAACAGATCACTTAAAGTTCTTCTGAAGCAACTTAAAGTTATCAGGCATGCATGGATCTTGGAGGAATCAGATGTGCAGTCAGG
GACCATAGCACAAGACAGGCGTCTTCTACTGGTGCTACCAGCAAATGCTGGAAGCCGGGAACACTGGGTACGTTGGAAACCACGTGATGTGAAGAAGTAAGAT
AAACTGTAGGAGAAAAGCATTTCGTAGTGGGCCATGAAGCCTTTCAGGACATGTATTGCAGTATGGGCCGGCCCATTACGCAATTGGACGACAACAAAGACTA
GTATTAGTACCACCTCGGCTATCCACATAGATCAAAGCTGATTTAAAAGAGTTGTGCAGATGATCCGTGGC

>pOsU6P2
GGATCATGAACCAACGGCCTGGCTGTATTTGGTGGTTGTGTAGGGAGATGGGGAGAAGAAAAGCCCGATTCTCTTCGCTGTGATGGGCTGGATGCATGCGGGG
GAGCGGGAGGCCCAAGTACGTGCACGGTGAGCGGCCCACAGGGCGAGTGTGAGCGCGAGAGGCGGGAGGAACAGTTTAGTACCACATTGCCCAGCTAACTCGA
ACGCGACCAACTTATAAACCCGCGCGCTGTCGCTTGTG

>pMtU6.6
ATGCCTATCTTATATGATCAATGAGGCATTTAATTGGGTGCATATGATGGTGAAAAAAGGTGCAGCTCCTGGCTTGGGAATGATGACTCATGTGGAATTTGGT
CTTAAATTTATCACATCCTTTTGGGATGTGATGATTGTATCACTTGTTCATTTTGCAAAGACAAGGTGCACTGCTACAAACTTTGGTTTAATCTGAAATAAAA
CAAAACTCACTGAGAGGAAGATGCATCCCAGTAGGTGAAAGTCGAGAAGGATTTGCATGTTACTATTACACTTGCTTTTTAGTCCCACATCGTCTGAAACATA
AAATATTTCAGCGTTTAAATACTTCAAGCGAACCAGTAGGCTT

>pZmU3
GGCGGCAGGGAGAGTTTTAACATTGACTAGCGTGCTGATAATTTGTGAGAAATAATAATTGACAAGTAGATACTGACATTTGAGAAGAGCTTCTGAACTGTTA
TTAGTAACAAAAATGGAAAGCTGATGCACGGAAAAAGGAAAGAAAAAGCCATACTTTTTTTTAGGTAGGAAAAGAAAAAGCCATACGAGACTGATGTCTCTCA
GATGGGCCGGGATCTGTCTATCTAGCAGGCAGCAGCCCTACCAACCTCACGGGCCAGCAATTACGAGTCCTTCTAAAACGTCCCGCCGAGGGCGCGTGGCCGT
GCTGTGCAGCAGCACGTCTAACATTAGTCCCACCTCGCCAGTTTACAGGGAGCAGAACCAGCTTATAAGCGGAGGCGCGGCACCAAGAAGC

>pTaU3
GGCGGCAGGGAGAGTTTTAACATTGACTAGCGTGCTGATAATTTGTGAGAAATAATAATTGACAAGTAGATACTGACATTTGAGAAGAGCTTCTGAACTGTTA
TTAGTAACAAAAATGGAAAGCTGATGCACGGAAAAAGGAAAGAAAAAGCCATACTTTTTTTTAGGTAGGAAAAGAAAAAGCCATACGAGACTGATGTCTCTCA
GATGGGCCGGGATCTGTCTATCTAGCAGGCAGCAGCCCTACCAACCTCACGGGCCAGCAATTACGAGTCCTTCTAAAACGTCCCGCCGAGGGCGCGTGGCCGT
GCTGTGCAGCAGCACGTCTAACATTAGTCCCACCTCGCCAGTTTACAGGGAGCAGAACCAGCTTATAAGCGGAGGCGCGGCACCAAGAAGC

>pTaU6
GACCAAGCCCGTTATTCTGACAGTTCTGGTGCTCAACACATTTATATTTATCAAGGAGCACATTGTTACTCACTGCTAGGAGGGAATCGAACTAGGAATATTG
ATCAGAGGAACTACGAGAGAGCTGAAGATAACTGCCCTCTAGCTCTCACTGATCTGGGCGCATAGTGAGATGCAGCCCACGTGAGTTCAGCAACGGTCTAGCG
CTGGGCTTTTAGGCCCGCATGATCGGGCTTTGTCGGGTGGTCGACGTGTTCACGATTGGGGAGAGCAACGCAGCAGTTCCTCTTAGTTTAGTCCCACCTCGCC
TGTCCAGCAGAGTTCTGACCGGTTTATAAACTCGCTTGCTGCATCAGACTTG

Yeast

>pSNR52
TCTTTGAAAAGATAATGTATGATTATGCTTTCACTCATATTTATACAGAAACTTGATGTTTTCTTTCGAGTATATACAAGGTGATTACATGTACGTTTGAAGT
ACAACTCTAGATTTTGTAGTGCCCTCTTGGGCTAGCGGTAAAGGTGCGCATTTTTTCACACCCTACAATGTTCTGTTCAAAAGATTTTGGTCAAACGCTGTAG
AAGTGAAAGTTGGTGCGCATGTTTCGGCGTTCGAAACTTCTCCGCAGTGAAAGATAAATGATC

Human

>phU6
GAGGGCCTATTTCCCATGATTCCTTCATATTTGCATATACGATACAAGGCTGTTAGAGAGATAATTAGAATTAATTTGACTGTAAACACAAAGATATTAGTAC
AAAATACGTGACGTAGAAAGTAATAATTTCTTGGGTAGTTTGCAGTTTTAAAATTATGTTTTAAAATGGACTATCATATGCTTACCGTAACTTGAAAGTATTT
CGATTTCTTGGCTTTATATATCTTGTGGAAAGGAC

Supplementary Figure S3. Analysis of promoters used for sgRNA expression in different organisms.
A total of ten promoters were examined for the presence of prokaryotic promoter features, such as for conserved -10 (TATAAT) and -35 (TTGACA) elements highlighted in green and cyan, respectively. Partially conserved nucleotide bases nearby -10 and -35 elements shaded in gray and pink, respectively. Seven sgRNA-expression plant-promoters include pAtU6 (Nekrasov et al., 2013), pOsU3 (Xing et al., 2014), pOsU6P2 (Lowder et al., 2015), pMtU6.6 (Jacobs et al., 2015), pZmU3 (Liang et al., 2014), pTaU3 (Lin et al., 2020), and pTaU6 (Shan et al., 2013). The sgRNA expression promoters for bacteria, yeast, and human consisted of pJ23119, pSNR52 (DiCarlo et al., 2013), and phU6 (Fu et al., 2013), respectively.

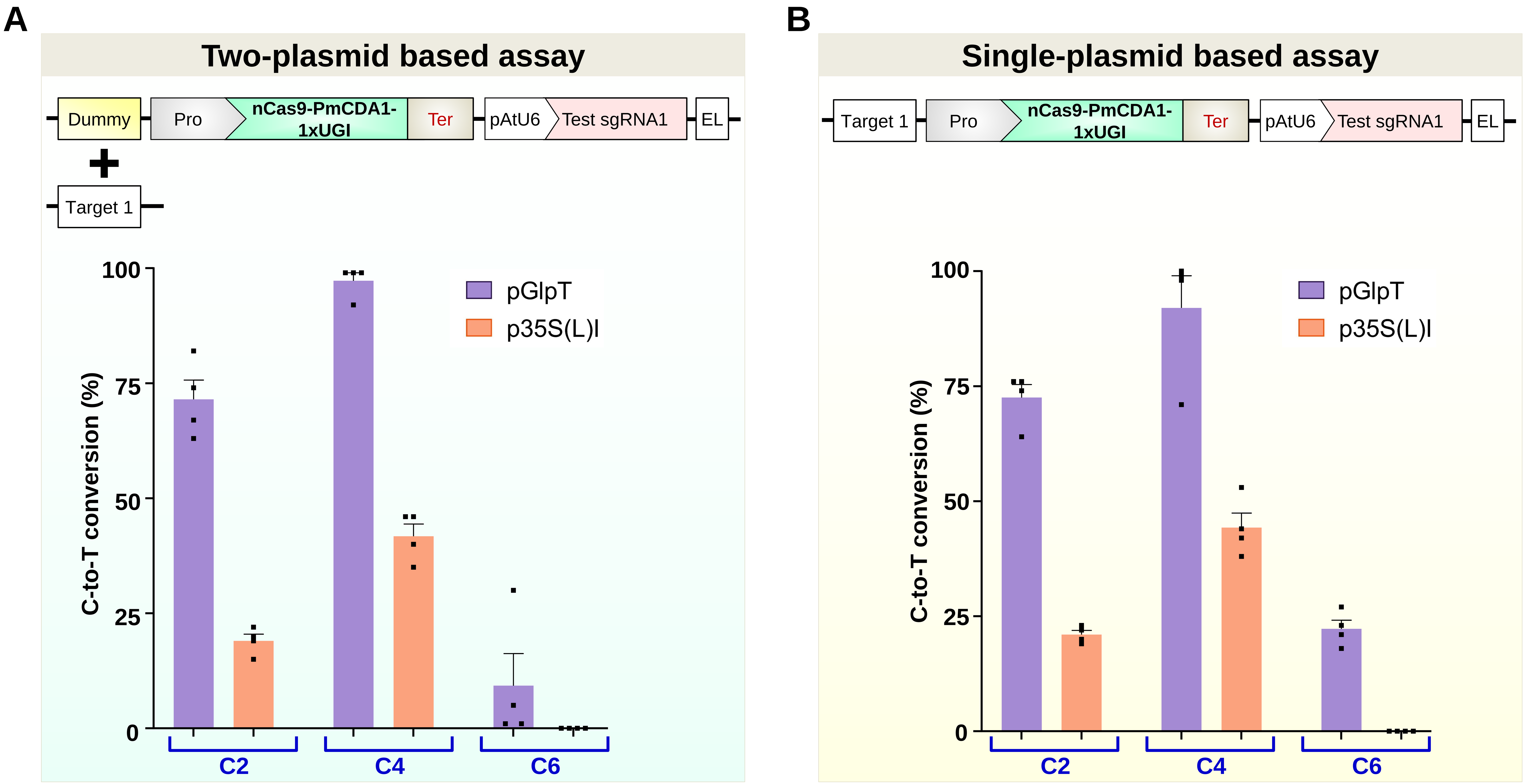

Supplementary Figure S4. Analysis of two-plasmid and single-plasmid vector systems for evaluation of C-to-T conversion by PmCDA1-mediated cytosine base editor. (A) Schematic representation of the two-plasmid vector system showing plasmid 1: two transcriptional units (TUs) composing of nCas9 (D10A) fused with PmCDA1-1xUGI and AtU6 promoter-sgRNA unit; and plasmid 2: a synthesized target region for sgRNA. Lower panel showing the PmCDA1-based C-to-T editing activities. (B) Single-plasmid system composed both the components in single vector, i.e., target region and two TUs for nCas9-BE and sgRNA (same data used for Figure 3C). Lower panel showing the PmCDA1-based C-to-T editing activities. The base conversion rate was estimated using the online tool EditR. Graph values show the mean percentage on the *y*-axis and the tested protospacer positions on the *x*-axis. The graph bar shows the mean of percentage values, and error bars indicate the standard error of the mean (mean ±s.e.m.) of four independent biological replicates. Dots indicate the individual biological replicates.

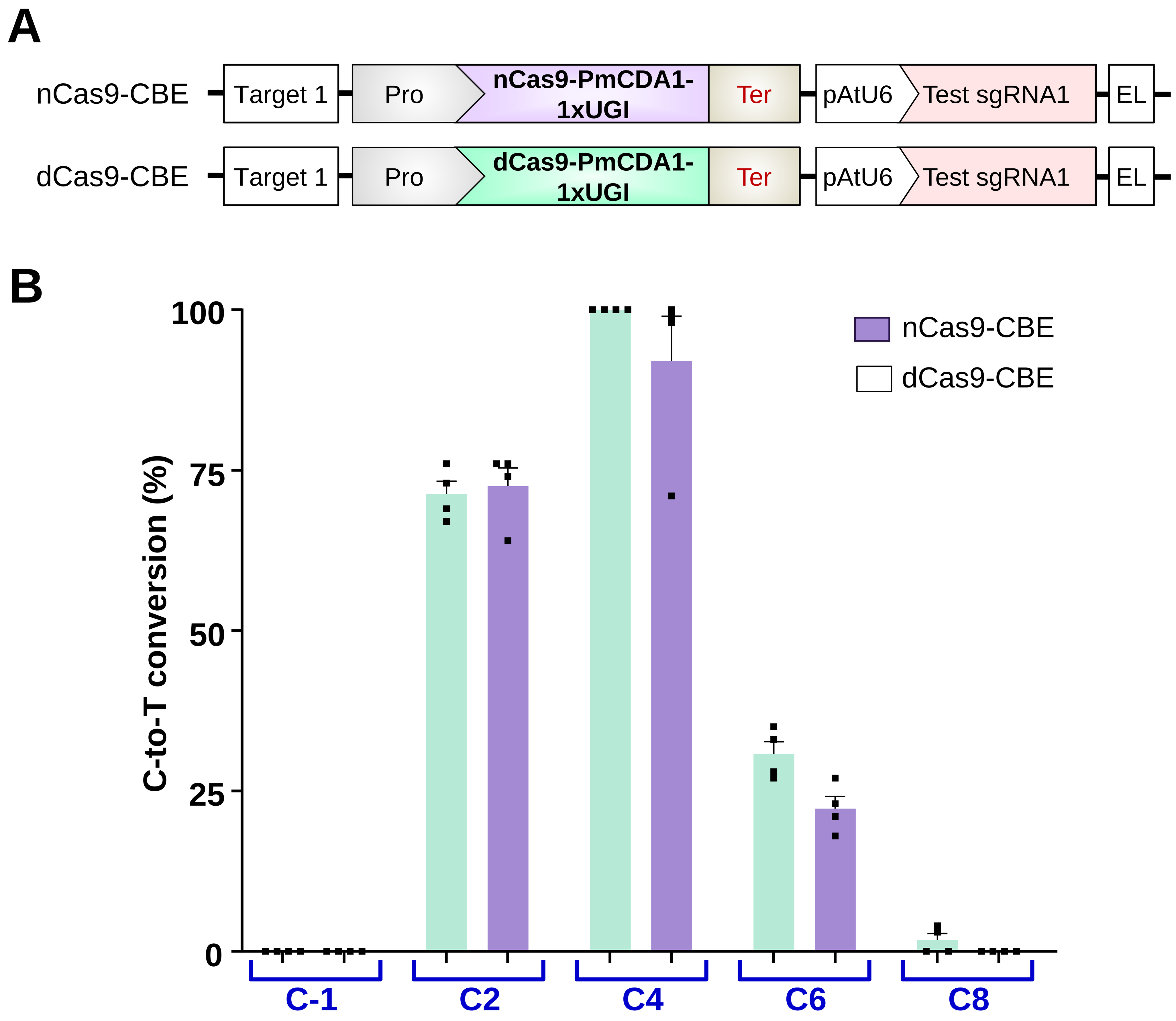

Supplementary Figure S5. C-to-T conversion by PmCDA1-mediated cytosine base editor designed using nickase (nCas9, D10A) and dead (dCas9, D10A+H840A) form of Cas9. (A) Schematic representation of the designed vectors with two transcriptional units (TUs) composing of nCas9 (D10A) or dCas9 (D10A+H840A) fused with PmCDA1-1xUGI and AtU6 promoter-sgRNA. (B) PmCDA1-based C-to-T editing activities. The base conversion rate was estimated using the online tool EditR. Graph values show the mean percentage on the *y*-axis and the tested protospacer positions on the *x*-axis. The graph bar shows the mean of percentage values, and error bars indicate the standard error of the mean (mean $\pm$s.e.m.) of four independent biological replicates. Dots indicate the individual biological replicates.

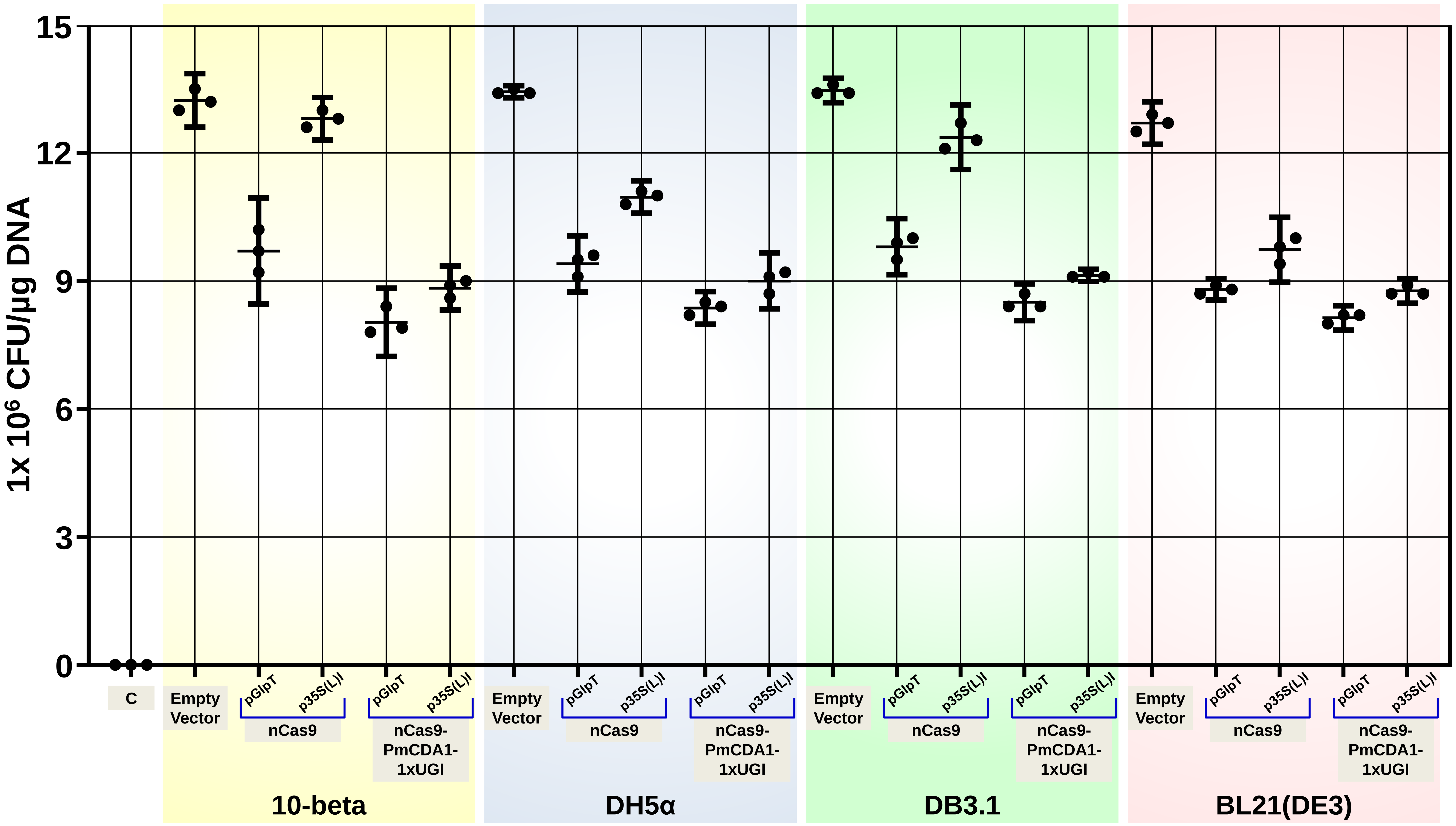

Supplementary Figure S6. Cytotoxicity assay of nCas9 and cytosine base editors in different *E. coli* strains. Plasmid vectors with *rppH* sgRNA3 (driven by pAtU6) targeting *rppH* gene in the genome were assembled containing combinations of nCas9(D10A) and nCas9(D10A)-PmCDA1-1xUGI expressed using either pGlpT or p35S(L)I promoters. Empty vector (pAGM4723) was used as non-BE control. C denotes the competent cells transformed with plasmid consisting BE components driven by pEc1 together with pJ23119-gRNA which showed no cell survival for all the tested strains. Equal amount of plasmid DNA was transformed into the competent *E. coli* cells of different strains. Viable cells were counted and calculated as colony forming unit (CFU) per amount of transformed plasmid DNA. Dots represent three independent experiments, and the line with error bar indicates the 95% confidence.

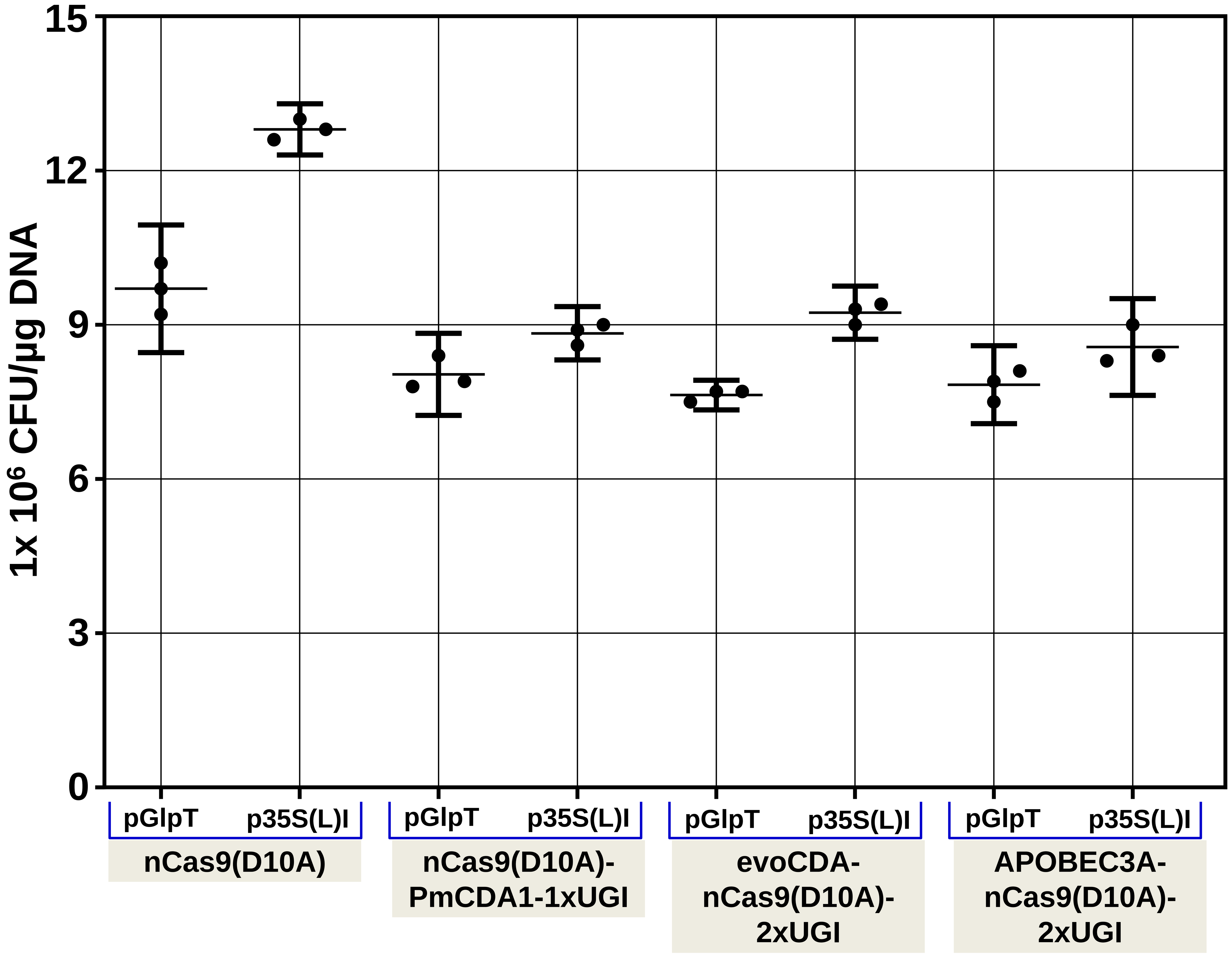

Supplementary Figure S7. Cytotoxicity assay of nCas9(D10A) and cytosine base editors.
Plasmid vectors with *rppH* sgRNA3 targeting *rppH* gene in the genome were assembled containing four combinations expressed using either pGlpT or p35S(L)I promoters [nCas9(D10A), nCas9(D10A)-PmCDA1-1xUGI, evoCDA1-nCas9(D10A)-2xUGI and APOBEC3A-nCas9(D10A)-2xUGI]. Equal amount of plasmid DNA was transformed into the competent 10-beta *E. coli* cells. Viable cells were counted and calculated as colony forming unit (CFU) per amount of transformed plasmid DNA. Dots represent three independent experiments, and the line with error bar indicates the 95% confidence

Inactive sgRNA1

GGTGAAGCAGCGGACAGCAGTGG

GGUGAAGCAGCGGACAGCAGGUUUUAGAGCUAGAAAUAGCAAGUUAAAAUAAGGCUAGUCCGUUAUCAACUUG
AAAAAGUGGCACCGAGUCGGUGCUUUU

Structure 1

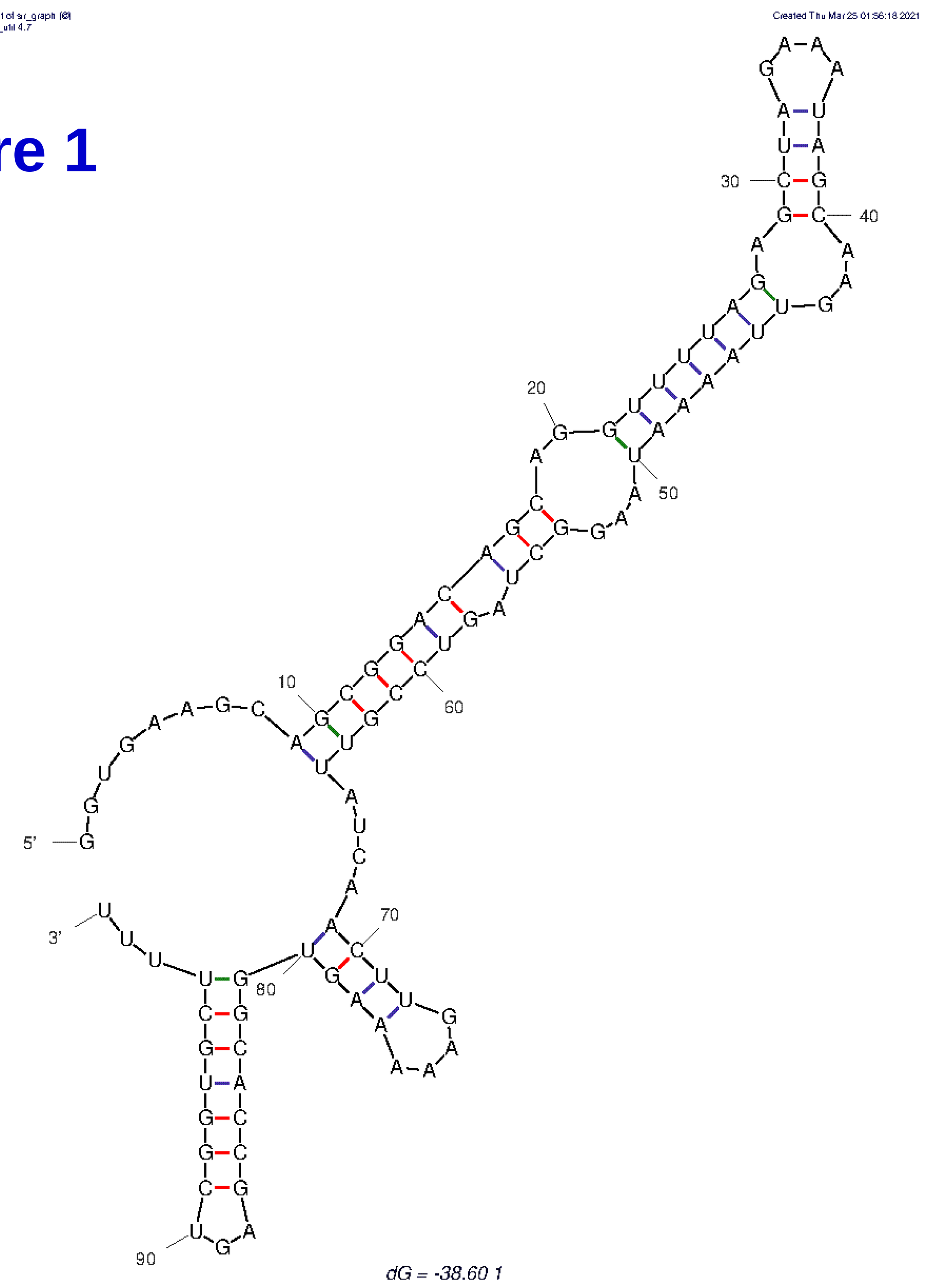

Structure 2

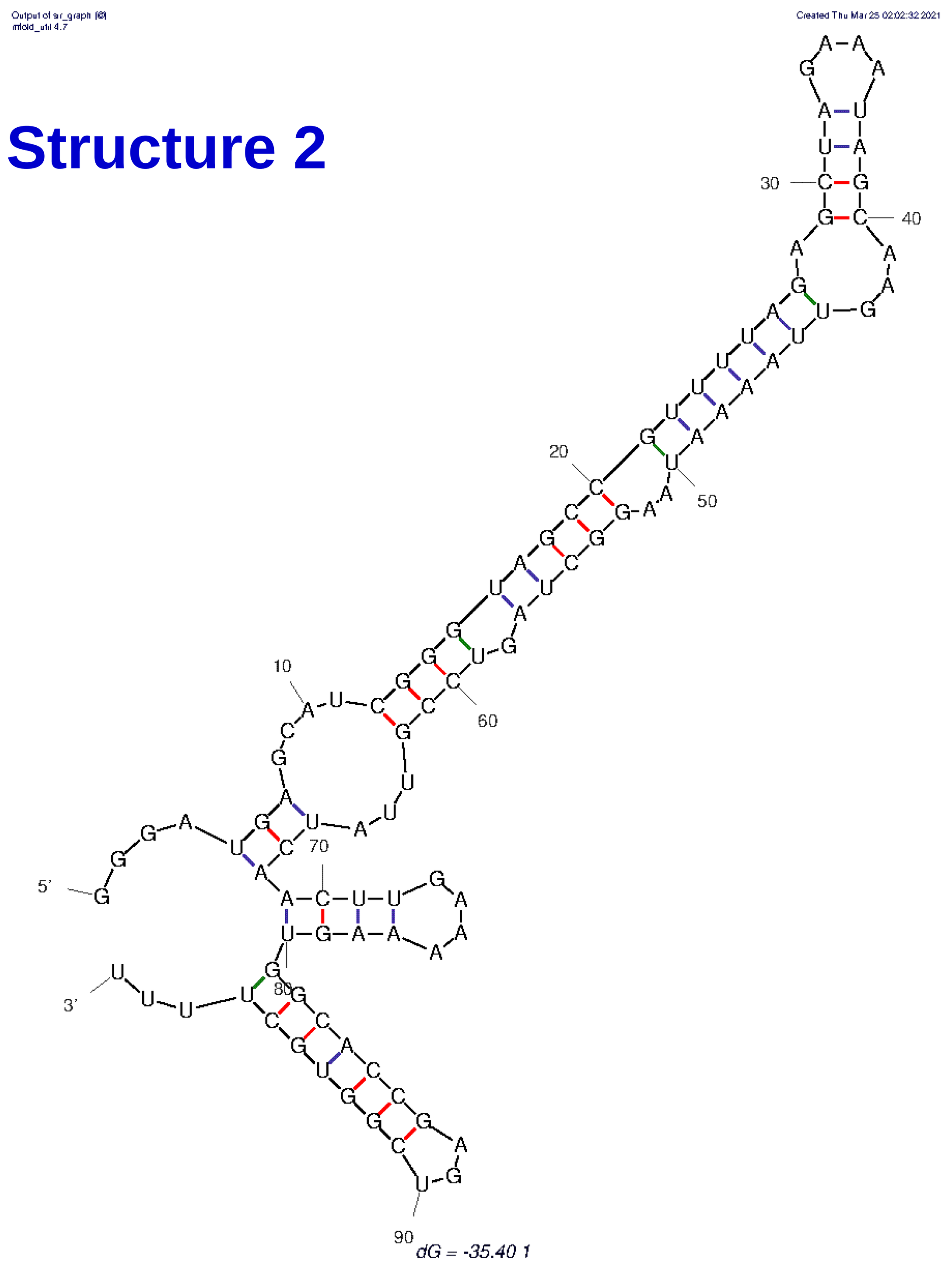

Inactive sgRNA2

GGGATGAGCATCGGGTAGCCTGG

GGGAUGAGCAUCGGGUAGCCGUUUUAGAG
CUAGAAAUAGCAAGUUAAAAUAAGGCUAG
UCCGUUAUCAACUUGAAAAAGUGGCACCG
AGUCGGUGCUUUU

Structure 1

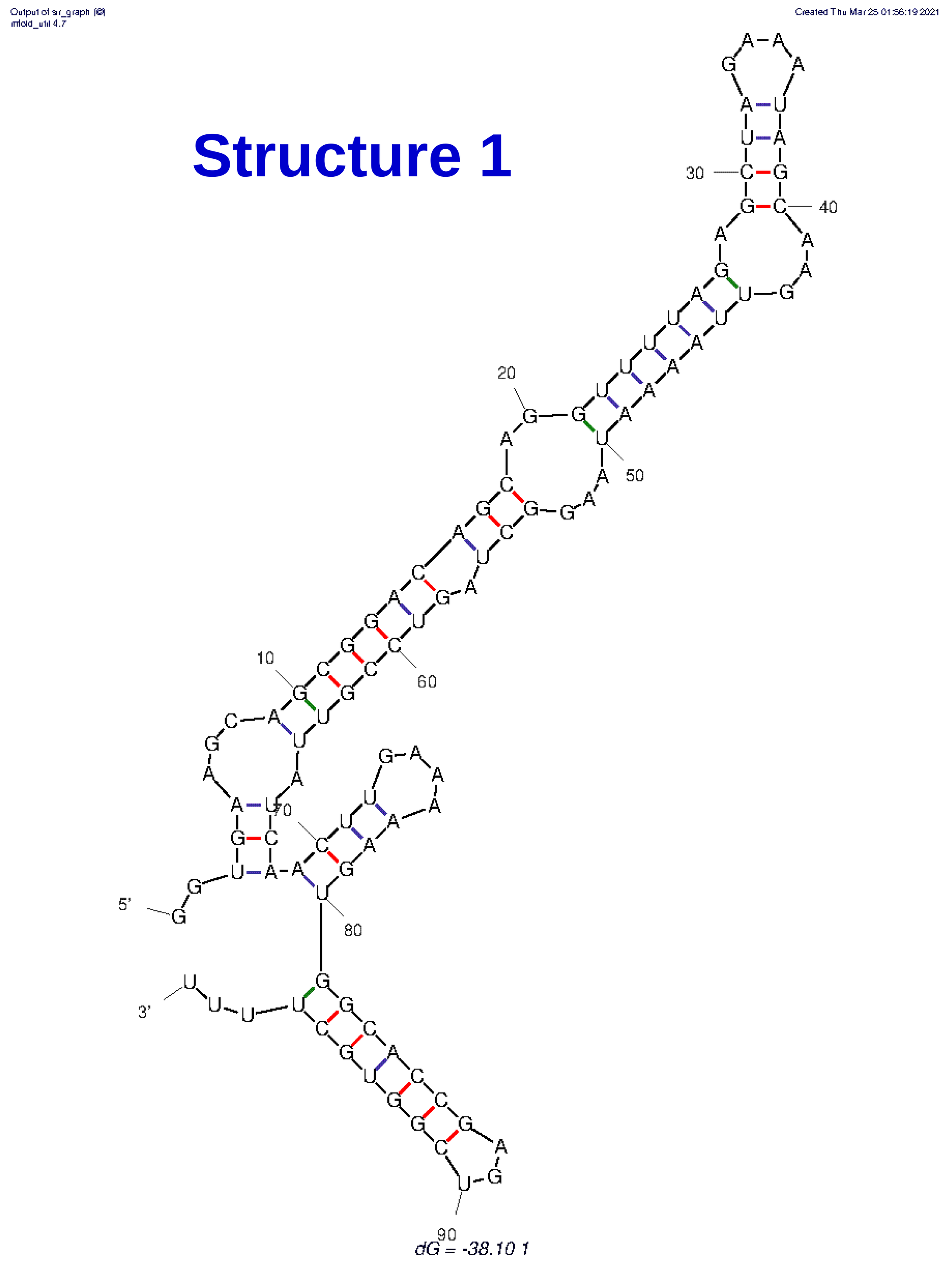

Supplementary Figure S8. Features of inactive sgRNAs from Figure 5.
Both the sgRNAs form stable interactions with the scaffold region that allow binding with Cas9, but it prevents target DNA recognition by the Cas9-sgRNA complex (Thyme et al., 2016). Secondary structures were predicted using the mfold v2.3 server using default parameters (Zuker 2003).

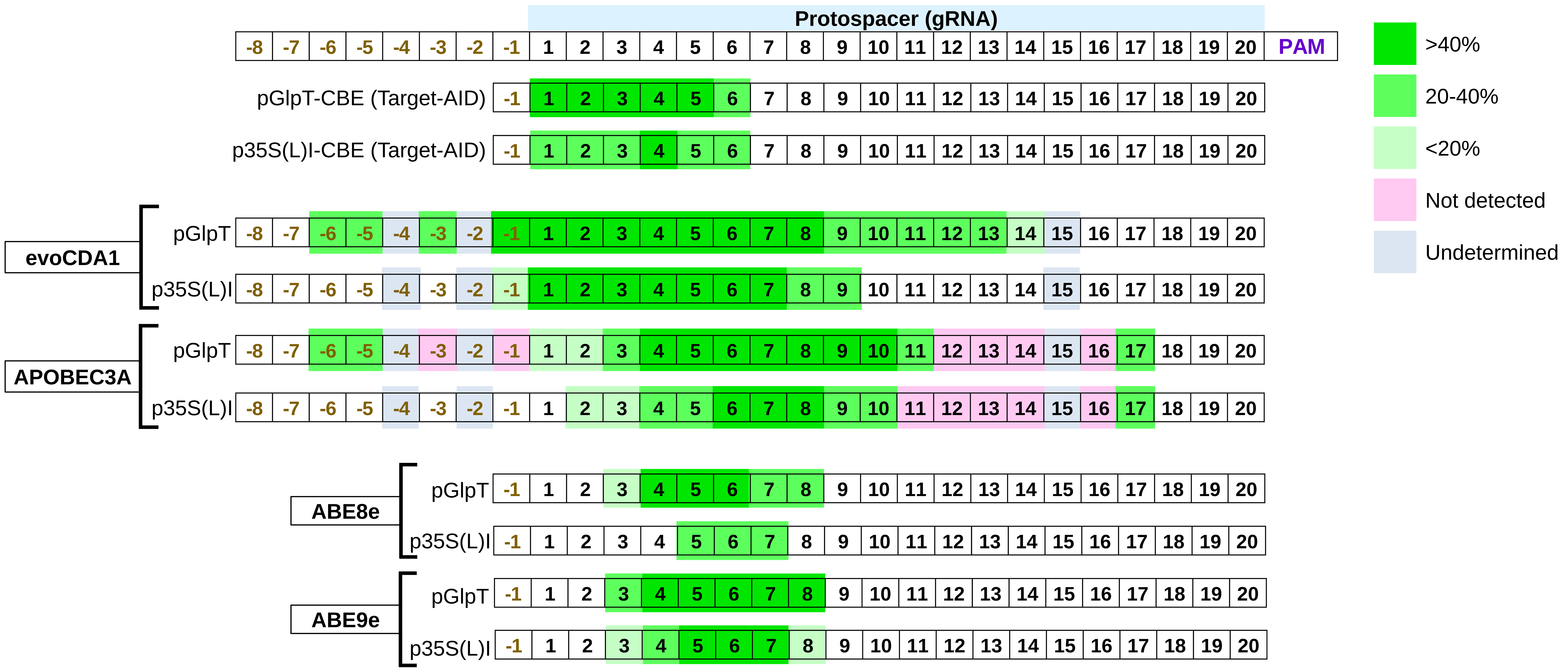

Supplementary Figure S9. Editing windows for CBEs (PmCDA1, evoCDA1, APOBEC3A) and ABEs (ABE8e, ABE9e) reported in the present study expressed under the promoters of different strengths.

Supplementary Table S1. Overview of cytotoxicity observed in different combinations of promoters tested for expression of BE components. (D10A) form of nCas9 was used in all the experiments. All the experiments were conducted in competent 10-beta *E. coli* cells. Test sgRNA1 and Test sgRNA2 -Target regions were targeted by respective sgRNAs and PmCDA1-based Target-AID system for C-to-T conversion expressed by chosen promoters. Promoter-BE reagent combinations exhibiting toxic effects on cell survival are highlighted in gray.

gRNA promoter	Cas9 type	BE Promoter	Construct Scheme	Cell survival
pJ23119	-	-	pJ23119-sgRNA	Yes
pAtU6	-	-	pAtU6-sgRNA	Yes
-	dCas9	pEc1	pEc1-dCas9-Link-PmCDA1-1xUGI	Yes
-	dCas9	pGlpT	pGlpT-dCas9-Link-PmCDA1-1xUGI	Yes
-	nCas9	pEc1	pEc1-nCas9-Link-PmCDA1-1xUGI	Yes
-	nCas9	pGlpT	pGlpT-nCas9-Link-PmCDA1-1xUGI	Yes
pJ23119	dCas9	pEc1	pEc1-dCas9-Link-PmCDA1-1xUGI + pJ23119-sgRNA	No
pJ23119	dCas9	pGlpT	pGlpT-dCas9-Link-PmCDA1-1xUGI + pJ23119-sgRNA	No
pJ23119	nCas9	pEc1	pEc1-nCas9-Link-PmCDA1-1xUGI + pJ23119-sgRNA	No
pJ23119	nCas9	pGlpT	pGlpT-nCas9-Link-PmCDA1-1xUGI + pJ23119-sgRNA	No
pAtU6	dCas9	pEc1	pEc1-dCas9-Link-PmCDA1-1xUGI + pAtU6-sgRNA	No
pAtU6	dCas9	pGlpT	pGlpT-dCas9-Link-PmCDA1-1xUGI + pAtU6-sgRNA	Yes
pAtU6	nCas9	pEc1	pEc1-nCas9-Link-PmCDA1-1xUGI + pAtU6-sgRNA	No*
pAtU6	nCas9	pGlpT	pGlpT-nCas9-Link-PmCDA1-1xUGI + pAtU6-sgRNA	Yes

* Only small number of colonies were obtained in repeated experiments that showed mix of correct and incorrect clones.

Supplementary Table S2. *Escherichia coli* strains used in the present study.

Strain	Genotype
10-beta	Δ(ara-leu) 7697 araD139 fhuA ΔlacX74 galK16 galE15 e14- ϕ80dlacZΔM15 recA1 relA1 endA1 nupG rpsL (StrR) rph spoT1 Δ(mrr-hsdRMS-mcrBC)
DH5α	F– φ80lacZΔ M15 Δ (lacZYA-argF) U169 recA1 endA1 hsdR17 (rK– mK+) phoA supE44 λ- thi–1 gyrA96 relA1
BL21(DE3)	fhuA2 [lon] ompT gal (λ DE3) [dcm] ΔhsdS λ DE3=λ sBamHIo ΔEcoRI-B int::(lacI::PlacUV5::T7 gene1) i21 Δnin5
DB3.1	gyrA462 endA1 Δ(sr1-recA) mcrB mrr hsdS20 glnV44 (=supE44) ara14 galK2 lacY1 proA2 rpsL20 xyl5 leuB6 mtl1

Supplementary Table S3. Primer sequences used for cloning and sequencing are summarized.

Name	Sequence (5'-3')	Description
**Protospacer oligos**		
Test gRNA1-F	TGTGGTCTCAATTGACACACACACTTAGAATCTGGTTTTAGAGCTAGAAATAGCAAGTTAAAAT	Test sgRNA1 PCR and cloning
Test gRNA2-F	TGTGGTCTCAATTGCACACACACATTAGAATCTGGTTTTAGAGCTAGAAATAGCAAGTTAAAAT	Test sgRNA2 PCR and cloning
Test gRNA3-F	TGTGGTCTCAATTGAAACCCCTCCCCCACCAGCGTTTTAGAGCTAGAAATAGCAAGTTAAAAT	Test sgRNA3 PCR and cloning
GL1-gRNA1-F	TGTGGTCTCAATTGGGAAAAGTTGTAGACTGAGAGTTTTAGAGCTAGAAATAGCAAGTTAAAAT	GL1-sgRNA1 PCR and cloning
SlMlo1-gRNA2-F	TGTGGTCTCAATTGTACAAAGTTAATCAAGAATGTTTTAGAGCTAGAAATAGCAAGTTAAAAT	SlMlo1-sgRNA2 PCR and cloning
SlPelo-gRNA1-F	TGTGGTCTCAATTGTCCAGCATCATTCAGTTGTGGTTTTAGAGCTAGAAATAGCAAGTTAAAAT	SlPelo-sgRNA1 PCR and cloning
Inactive gRNA1-F	TGTGGTCTCAATTGGGTGAAGCAGCGGACAGCAGGTTTTAGAGCTAGAAATAGCAAGTTAAAAT	Inactive sgRNA1 PCR and cloning
Inactive gRNA2-F	TGTGGTCTCAATTGGGGATGAGCATCGGGTAGCCGTTTTAGAGCTAGAAATAGCAAGTTAAAAT	Os- sgRNA22 PCR and cloning
Os- gRNA22-F	TGTGGTCTCAATTGCTTAATTTCTGTTAGGTTCTGTTTTAGAGCTAGAAATAGCAAGTTAAAAT	Os- sgRNA23 PCR and cloning
Os- gRNA23-F	TGTGGTCTCAATTGAGTACAAGTTTATTTTTTTCGTTTTAGAGCTAGAAATAGCAAGTTAAAAT	*galK* sgRNA1 PCR and cloning
galK gRNA1-F	TGTGGTCTCAATTGCAACTGCGTAACAACAGCTTGTTTTAGAGCTAGAAATAGCAAGTTAAAAT	*rpoB* sgRNA1 PCR and cloning
rpoB gRNA1-F	TGTGGTCTCAATTGGGTCCATAAACTGAGACAGCGTTTTAGAGCTAGAAATAGCAAGTTAAAAT	*rppH* sgRNA1 PCR and cloning
rppH gRNA1-F	TGTGGTCTCAATTGATCGCCAGGGGCAGGTAATGGTTTTAGAGCTAGAAATAGCAAGTTAAAAT	*rppH* sgRNA2 PCR and cloning
rppH gRNA2-F	TGTGGTCTCAATTGAGGGGCAGGTAATGTGGGCCGTTTTAGAGCTAGAAATAGCAAGTTAAAAT	*rppH* sgRNA3 PCR and cloning
rppH gRNA3-F	TGTGGTCTCAATTGTCCTGGCAATTTCCGCAAGGGTTTTAGAGCTAGAAATAGCAAGTTAAAAT	PCR and cloning
sgRNA Rev	TGTGGTCTCAAGCGTAATGCCAACTTTGTAC	sgRNA PCR and cloning
**Target oligos**		
Target (Test gRNA1)-F	CAGTCCGTCTCTTTCGTCAAGTGCGACTCCCCATCGACACACACACACACTTAGAATATGGGGAGCTTTGAGACGGACT	Target (Test sgRNA1)
Target (Test gRNA1)-R	AGTCCGTCTCAAAGCTCCCCATATTCTAAGTGTGTGTGTGTGTCGATGGGGAGTCGCACTTGACGAAAGAGACGGACTG	Target (Test sgRNA1)
Target (Test gRNA2)-F	CAGTCCGTCTCTTTCGTCAAGTGCGACTCCCCATCGACACCACACACACATTAGAATCTGGGGAGCTTTGAGACGGACT	Target (Test sgRNA2)

Target (Test gRNA2)-R	AGTCCGTCTCAAAGCTCCCCAGATTCTAATGTGTGTGTGGTGTCGATGGG GAGTCGCACTTGACGAAAGAGACGGACTG	Target (Test sgRNA2)
Target (Test gRNA3)-F	CAGTCCGTCTCTTTCGTCAAGTGCGACTCCCCATCGACACAAACCCCTCC CCCCACCAGCCGGAGCTTTGAGACGGACT	Target (Test sgRNA3)
Target (Test gRNA3)-R	AGTCCGTCTCAAAGCTCCGGCTGGTGGGGGGAGGGGTTTGTGTCGATGG GGAGTCGCACTTGACGAAAGAGACGGACTG	Target (Test sgRNA3)
Target (AtGL1-gRNA1)-F	CAGTCGAAGACAATAAGGGAAAAGTTGTAGACTGAGATGGAAGTGTTGT CTTCGACTG	Target (AtGL1-sgRNA1)
Target (AtGL1-gRNA1)-R	CAGTCGAAGACAATAAGGATCAAAGACCATCTGAATTTGGAAGTGTTGTC TTCGACTG	Target (AtGL1-sgRNA1)
Target (SlMlo1-gRNA2)-F	CAGTCCGTCTCTTTCGGAATTGTACAAAGTTAATCAAGAATAGGGGGCTT TGAGACGGACT	Target (SlMlo1-sgRNA2)
Target (SlMlo1-gRNA2)-R	AGTCCGTCTCAAAGCCCCCTATTCTTGATTAACTTTGTACAATTCCGAAAG AGACGGACTG	Target (SlMlo1-sgRNA2)
Target (SlPelo-gRNA1)-F	CAGTCCGTCTCTTTCGATATATTAGACATCCAGCATCATTCAGTTGTGGGG AGCTTTGAGACGGACT	Target (SlPelo-sgRNA1)
Target-(SlPelo-gRNA1)-R	AGTCCGTCTCAAAGCTCCCCACAACTGAATGATGCTGGATGTCTAATATAT CGAAAGAGACGGACTG	Target (SlPelo-sgRNA1)
Target-(Inactive gRNA1)-F	CAGTCCGTCTCTTTCGCCCCATCGACACGGTGAAGCAGCGGACAGCAGT GGAGCTTTGAGACGGACT	Target-(Inactive sgRNA1)
Target-(Inactive gRNA1)-R	AGTCCGTCTCAAAGCTCCACTGCTGTCCGCTGCTTCACCGTGTCGATGGG GCGAAAGAGACGGACTG	Target-(Inactive sgRNA1)
Target-(Inactive gRNA2)-F	CAGTCCGTCTCTTTCGCCCCATCGACACGGGATGAGCATCGGGTAGCCTG GAGCTTTGAGACGGACT	Target-(Inactive sgRNA2)
Target-(Inactive gRNA2)-R	AGTCCGTCTCAAAGCTCCAGGCTACCCGATGCTCATCCCGTGTCGATGGG GCGAAAGAGACGGACTG	Target-(Inactive sgRNA2)
Target-(Os- gRNA22)-F	CAGTCCGTCTCTTTCGCTAATTCATGGACTTAATTTCTGTTAGGTTCTTGG AGCTTTGAGACGGACT	Target-(Os- sgRNA22)
Target-(Os- gRNA22)-R	AGTCCGTCTCAAAGCTCCAAGAACCTAACAGAAATTAAGTCCATGAATTA GCGAAAGAGACGGACTG	Target-(Os- sgRNA22)
Target-(Os- gRNA23)-F	CAGTCCGTCTCTTTCGATGGCATACTGAAGTACAAGTTTATTTTTTTCTGG AGCTTTGAGACGGACT	Target-(Os- sgRNA23)
Target-(Os- gRNA23)-R	AGTCCGTCTCAAAGCTCCAGAAAAAAATAAACTTGTACTTCAGTATGCCA TCGAAAGAGACGGACTG	Target-(Os- sgRNA23)
**qRT-PCR**		
qSfGFP-F	GGTGAAGGTGACGCAACTAA	qRT-PCR of *sfGFP*
qSfGFP-R	GCAAAGCACTGAACACCATAAG	qRT-PCR of *sfGFP*
qRrsA-F	CTCTTGCCATCGGATGTGCCCA	qRT-PCR of 16S rRNA
qRrsA-R	CCAGTGTGGCTGGTCATCCTCTCA	qRT-PCR of 16S rRNA
**Target PCR and Sanger Sequencing**		
L1-F1	GATGGGCTGCCTGTATCGAGT	Target region seq
galk9-F	GCCAACGCATTTGGCTACCCTG	*galK* PCR and seq

galk9-R	CATGCGCAACAGCGTTGAACTC	*galK* PCR
rpoB-F	CCTCGGCAACCGTCGTATCC	*rpoB* PCR and seq
rpoB-R	CCTGGGCGATAACGTAGTTGC	*rpoB* PCR
rpph-F	CGGCTATCCACCCCTTCCTCTG	*rppH* PCR and seq
rpph-R1	GATTTCTGCATCGCCGCTCACC	*rppH* PCR
**PCR**		
pGlpT-F1	GAGAAGACTTGGAGGAAAGTGAAACGTGATTTCATGCGTC	pGlpT (Pro-5U) PCR
pGlpT-R1	ACTGAAGACAACATTCTAGTATTTCTCCTCTTTCTCTAG	pGlpT (Pro-5U) PCR
pGlpT-R2	ACTGAAGACAAATGGCTAGTATTTCTCCTCTTTCTCTAG	pGlpT (Pro-5Uf) PCR
Flag-F1	ACTGAAGACTTAATGGACTATAAGGACCACGACGGAGACTACAAGGATCATG ATATTGATTACAAAGACGATGACGATAAGGTTTCGAAGTCTTCACTG	Design of universal target acceptor
Flag-R1	CAGTGAAGACTTCGAAACCTTATCGTCATCGTCTTTGTAATCAATATCATGATC CTTGTAGTCTCCGTCGTGGTCCTTATAGTCCATTAAGTCTTCAGT	Design of universal target acceptor
DummyCT-F1	ACTGAAGACTTTTCGCGAGACGAGTAAGAATTCTAGTGAGGGCGTCTCTGCT TAAGTCTTCACTG	Design of universal target acceptor
DummyCT-R1	CAGTGAAGACTTAAGCAGAGACGCCCTCACTAGAATTCTTACTCGTCTCGCG AAAAGTCTTCAGT	Design of universal target acceptor
sfGFP-F1	GAGAAGACTTGCTTCTCGTAAAGGCGAAGAGCTGTTCACTGGT	Design of universal target acceptor
sfGFP-R1	ACTGAAGACAAAGCGTATAAACGCAGAAAGGCCCACCCGAA	Design of universal target acceptor
L3S2P2-F1	GAGAAGACTTGCTTCTCGGTACCAAATTCCAGAAAAGAGGCCTCCCGAAAG GGGGGCCTTTTTTCGTTTTGGTCCCGCTAAGTCTTCACT	TerL3S2P21 (3U-Ter) cloning
L3S2P2-R1	AGTGAAGACTTAGCGGGACCAAAACGAAAAAAGGCCCCCCTTTCGGGAGGC CTCTTTTCTGGAATTTGGTACCGAGAAGCAAGTCTTCTC	TerL3S2P21 (3U-Ter) cloning
eCDA-F1	GCATGAAGACTTCCATGAAACGGACAGCCGACGGAAGCGAGTTCGAGTCA	evoCDA domestication (NT1)
eCDA-R1	CAGTGAAGACTTGCTCTCTTCAGTGTTTTCTCCAGCCACCGATTC	evoCDA domestication (NT1)
eCDA-F2	GCATGAAGACTTGAGCCGAGAAGCGGAGAAGCGA	evoCDA domestication (NT1)
eCDA-R2	CAGTGAAGACTTCATTCCGCTGCCGCCGCTGCTGCCGCCACT	evoCDA domestication (NT1)
2xU-F1	GCATGAAGACTTGCTTCTAGCGGGGGGAGCACTAATCTGAGCGACA	2xUGI cloning
2xU-R1	CAGTGAAGACTTTACCTTAGACTTTCCTCTTCTTCTTGGGCTCGA	2xUGI cloning
A3A-F1	GCATGAAGACTTCCATGAAACGGACAGCCGACGGAAGCGAGTTCGAGTCAC CAAAGAAGAAGCGGAAAGTCGAGGCCAGCCCGGCTAGCGGCCCAAGG	APOBEC3A cloning (NT1)
A3A-R1	CAGTGAAGACTTCATTCCCTTAAGAGATTCTGGGGTGGCCGAC	APOBEC3A cloning (NT1)

Supplementary Table S4. Plasmids used in the present work.

**Name**	**Feature**	**Source**
pICH86966::AtU6p::sgRNA_PDS	PCR template for sgRNA	Nekrasov et al., 2013
pYTK001 (Addgene #65108)	PCR template for pGlpT, sfGFP, and sfGFP-Ter	Lee et al., 2015
pICSL01009::AtU6p	Source of AtU6 promoter	Nekrasov et al., 2013
pEc1-sfGFP-TerL3S2P21	L1 module for promoter activity analysis	This work
pGlpT-sfGFP-TerL3S2P21	L1 module for promoter activity analysis	This work
p35S(L)I-sfGFP-Ter35S	L1 module for promoter activity analysis	This work
pICH51266 (Addgene #50267)	Source of p35S(L)	Engler et al., 2014
pICH51277 (Addgene #50268)	Source of p35S(S)	Engler et al., 2014
pICH45195 (Addgene #50275)	Source of pRbc	Engler et al., 2014
pICH41414 (Addgene #50337)	Source of Ter35S	Engler et al., 2014
pKI1.1R (Addgene #85808)	Source of pRPS5a and TerHSP	Tsutsui and Higashiyama, 2017
Level 1 hCas9 module (Addgene #49771)	Source of Cas9	Nekrasov et al., 2013
pGlpT-nCas9-PmCDA1-1xUGI-TerL3S2P21	L1 module for Target-AID-based C-to-T editing	This work
p35S(L)I-nCas9-PmCDA1-1xUGI-Ter35S	L1 module for Target-AID-based C-to-T editing	This work
p35S(S)-nCas9-PmCDA1-1xUGI-Ter35S	L1 module for Target-AID-based C-to-T editing	This work
p35S(L)-nCas9-PmCDA1-1xUGI-Ter35S	L1 module for Target-AID-based C-to-T editing	This work
pRbc-nCas9-PmCDA1-1xUGI-Ter35S	L1 module for Target-AID-based C-to-T editing	This work
pRPS5a-sfGFP-PmCDA1-1xUGI-TerHSP	L1 module for Target-AID-based C-to-T editing	This work
PmCDA1-1xUGI (Addgene #79620)	Source of PmCDA1-1xUGI	Nishida et al., 2016
evoCDA1 pBT277 (Addgene #122608)	Source of evoCDA1 and 2xUGI	Thuronyi et al., 2019
A3A-PBE-ΔUGI (Addgene #119770)	Source of APOBEC3A	Zong et al., 2018
pGlpT-evoCDA1-nCas9-2xUGI-TerL3S2P21	L1 module for evoCDA1-based C-to-T editing	This work
p35S(L)I-evoCDA1-nCas9-2xUGI-Ter35S	L1 module for evoCDA1-based C-to-T editing	This work
pGlpT-A3A-nCas9-2xUGI-TerL3S2P21	L1 module for APOBEC3A-based C-to-T editing	This work
p35S(L)I-A3A-nCas9-2xUGI-Ter35S	L1 module for APOBEC3A-based C-to-T editing	This work
pGlpT-ABE8e-nCas9-TerL3S2P21	L1 module for ABE8e-based A-to-G editing	This work
p35S(L)I-ABE8e-nCas9-Ter35S	L1 module for ABE8e-based A-to-G editing	This work

Supplementary Table S5. DNA sequences of CRISPR and BE components

>nCas9 (D10A)

ATGGATAAAAAGTATTCTATTGGTTTAGCCATCGGCACTAATTCCGTTGGATGGGCTGTCATAACCGATGAATACAAAGTACCTTCAAAGAAATTT
AAGGTGTTGGGGAACACAGACCGTCATTCGATTAAAAAGAATCTTATCGGTGCCCTCCTATTCGATAGTGGCGAAACGGCAGAGGCGACTCGCC
TGAAACGAACCGCTCGGAGAAGGTATACACGTCGCAAGAACCGAATATGTTACTTACAAGAAATTTTTAGCAATGAGATGGCCAAAGTTGACG
ATTCTTTCTTTCACCGTTTGGAAGAGTCCTTCCTTGTCGAAGAGGACAAGAAACATGAACGGCACCCCATCTTTGGAAACATAGTAGATGAGGT
GGCATATCATGAAAAGTACCCAACGATTTATCACCTCAGAAAAAAGCTAGTTGACTCAACTGATAAAGCGGACCTGAGGTTAATCTACTTGGCTC
TTGCCCATATGATAAAGTTCCGTGGGCACTTTCTCATTGAGGGTGATCTAAATCCGGACAACTCGGATGTCGACAAACTGTTCATCCAGTTAGTA
CAAACCTATAATCAGTTGTTTGAAGAGAACCCTATAAATGCAAGTGGCGTGGATGCGAAGGCTATTCTTAGCGCCCGCCTCTCTAAATCCCGACG
GCTAGAAAACCTGATCGCACAATTACCCGGAGAGAAGAAAAATGGGTTGTTCGGTAACCTTATAGCGCTCTCACTAGGCCTGACACCAAATTTT
AAGTCGAACTTCGACTTAGCTGAAGATGCCAAATTGCAGCTTAGTAAGGACACGTACGATGACGATCTCGACAATCTACTGGCACAAATTGGAG
ATCAGTATGCGGACTTATTTTTGGCTGCCAAAAACCTTAGCGATGCAATCCTCCTATCTGACATACTGAGAGTTAATACTGAGATTACCAAGGCGC
CGTTATCCGCTTCAATGATCAAAAGGTACGATGAACATCACCAAGACTTGACACTTCTCAAGGCCCTAGTCCGTCAGCAACTGCCTGAGAAATA
TAAGGAAATATTCTTTGATCAGTCGAAAAACGGGTACGCAGGTTATATTGACGGCGGAGCGAGTCAAGAGGAATTCTACAAGTTTATCAAACCC
ATATTAGAGAAGATGGATGGGACGGAAGAGTTGCTTGTAAAACTCAATCGCGAAGATCTACTGCGAAAGCAGCGGACTTTCGACAACGGTAGC
ATTCCACATCAAATCCACTTAGGCGAATTGCATGCTATACTTAGAAGGCAGGAGGATTTTTATCCGTTCCTCAAAGACAATCGTGAAAAGATTGA
GAAAATCCTAACCTTTCGCATACCTTACTATGTGGGACCCCTGGCCCGAGGGAACTCTCGGTTCGCATGGATGACAAGAAAGTCCGAAGAAACG
ATTACTCCATGGAATTTTGAGGAAGTTGTCGATAAAGGTGCGTCAGCTCAATCGTTCATCGAGAGGATGACCAACTTTGACAAGAATTTACCGA
ACGAAAAAGTATTGCCTAAGCACAGTTTACTTTACGAGTATTTCACAGTGTACAATGAACTCACGAAAGTTAAGTATGTCACTGAGGGCATGCG
TAAACCCGCCTTTCTAAGCGGAGAACAGAAGAAAGCAATAGTAGATCTGTTATTCAAGACCAACCGCAAAGTGACAGTTAAGCAATTGAAAGA
GGACTACTTTAAGAAAATTGAATGCTTCGATTCTGTCGAGATCTCCGGGGTAGAAGATCGATTTAATGCGTCACTTGGTACGTATCATGACCTCCT
AAAGATAATTAAAGATAAGGACTTCCTGGATAACGAAGAGAATGAAGATATCTTAGAAGATATAGTGTTGACTCTTACCCTCTTTGAAGATCGGG
AAATGATTGAGGAAAGACTAAAAACATACGCTCACCTGTTCGACGATAAGGTTATGAAACAGTTAAAGAGGCGTCGCTATACGGGCTGGGGAC
GATTGTCGCGGAAACTTATCAACGGGATAAGAGACAAGCAAAGTGGTAAAACTATTCTCGATTTTCTAAAGAGCGACGGCTTCGCCAATAGGAA
CTTTATGCAGCTGATCCATGATGACTCTTTAACCTTCAAAGAGGATATACAAAAGGCACAGGTTTCCGGACAAGGGGACTCATTGCACGAACAT
ATTGCGAATCTTGCTGGTTCGCCAGCCATCAAAAAGGGCATACTCCAGACAGTCAAAGTAGTGGATGAGCTAGTTAAGGTCATGGGACGTCACA
AACCGGAAAACATTGTAATCGAGATGGCACGCGAAAATCAAACGACTCAGAAGGGGCAAAAAAACAGTCGAGAGCGGATGAAGAGAATAGA
AGAGGGTATTAAAGAACTGGGCAGCCAGATCTTAAAGGAGCATCCTGTGGAAAATACCCAATTGCAGAACGAGAAACTTTACCTCTATTACCTA
CAAAATGGAAGGGACATGTATGTTGATCAGGAACTGGACATAAACCGTTTATCTGATTACGACGTCGATCACATTGTACCCCAATCCTTTTTGAA
GGACGATTCAATCGACAATAAAGTGCTTACACGCTCGGATAAGAACCGAGGGAAAAGTGACAATGTTCCAAGCGAGGAAGTCGTAAAGAAAAT
GAAGAACTATTGGCGGCAGCTCCTAAATGCGAAACTGATAACGCAAAGAAAGTTCGATAACTTAACTAAAGCTGAGAGGGGTGGCTTGTCTGA
ACTTGACAAGGCCGGATTTATTAAACGTCAGCTCGTGGAAACCCGCCAAATCACAAAGCATGTTGCACAGATACTAGATTCCCGAATGAATACG
AAATACGACGAGAACGATAAGCTGATTCGGGAAGTCAAAGTAATCACTTTAAAGTCAAAATTGGTGTCGGACTTCAGAAAGGATTTTCAATTCT
ATAAAGTTAGGGAGATAAATAACTACCACCATGCGCACGACGCTTATCTTAATGCCGTCGTAGGGACCGCACTCATTAAGAAATACCCGAAGCTA
GAAAGTGAGTTTGTGTATGGTGATTACAAAGTTTATGACGTCCGTAAGATGATCGCGAAAAGCGAACAGGAGATAGGCAAGGCTACAGCCAAA
TACTTCTTTTATTCTAACATTATGAATTTCTTTAAGACGGAAATCACTCTGGCAAACGGAGAGATACGCAAACGACCTTTAATTGAAACCAATGG
GGAGACAGGTGAAATCGTATGGGATAAGGGCCGGGACTTCGCGACGGTGAGAAAAGTTTTGTCCATGCCCCAAGTCAACATAGTAAAGAAAAC
TGAGGTGCAGACCGGAGGGTTTTCAAAGGAATCGATTCTTCCAAAAAGGAATAGTGATAAGCTCATCGCTCGTAAAAAGGACTGGGACCCGAA
AAAGTACGGTGGCTTCGATAGCCCTACAGTTGCCTATTCTGTCCTAGTAGTGGCAAAAGTTGAGAAGGGAAAATCCAAGAAACTGAAGTCAGT
CAAAGAATTATTGGGGATAACGATTATGGAGCGCTCGTCTTTTGAAAAGAACCCCATCGACTTCCTTGAGGCGAAAGGTTACAAGGAAGTAAAA
AAGGATCTCATAATTAAACTACCAAAGTATAGTCTGTTTGAGTTAGAAAATGGCCGAAAACGGATGTTGGCTAGCGCCGGAGAGCTTCAAAAGG
GGAACGAACTCGCACTACCGTCTAAATACGTGAATTTCCTGTATTTAGCGTCCCATTACGAGAAGTTGAAAGGTTCACCTGAAGATAACGAACA
GAAGCAACTTTTTGTTGAGCAGCACAAACATTATCTCGACGAAATCATAGAGCAAATTTCGGAATTCAGTAAGAGAGTCATCCTAGCTGATGCC
AATCTGGACAAAGTATTAAGCGCATACAACAAGCACAGGGATAAACCCATACGTGAGCAGGCGGAAAATATTATCCATTTGTTTACTCTTACCAA
CCTCGGCGCTCCAGCCGCATTCAAGTATTTTGACACAACGATAGATCGCAAACGATACACTTCTACCAAGGAGGTGCTAGACGCGACACTGATT
CACCAATCCATCACGGGATTATATGAAACTCGGATAGATTTGTCACAGCTTGGGGGTGAC

>dCas9 (D10A+H140A)

ATGGATAAAAAGTATTCTATTGGTTTAGCCATCGGCACTAATTCCGTTGGATGGGCTGTCATAACCGATGAATACAAAGTACCTTCAAAGAAATTT
AAGGTGTTGGGGAACACAGACCGTCATTCGATTAAAAAGAATCTTATCGGTGCCCTCCTATTCGATAGTGGCGAAACGGCAGAGGCGACTCGCC
TGAAACGAACCGCTCGGAGAAGGTATACACGTCGCAAGAACCGAATATGTTACTTACAAGAAATTTTTAGCAATGAGATGGCCAAAGTTGACG
ATTCTTTCTTTCACCGTTTGGAAGAGTCCTTCCTTGTCGAAGAGGACAAGAAACATGAACGGCACCCCATCTTTGGAAACATAGTAGATGAGGT
GGCATATCATGAAAAGTACCCAACGATTTATCACCTCAGAAAAAAGCTAGTTGACTCAACTGATAAAGCGGACCTGAGGTTAATCTACTTGGCTC
TTGCCCATATGATAAAGTTCCGTGGGCACTTTCTCATTGAGGGTGATCTAAATCCGGACAACTCGGATGTCGACAAACTGTTCATCCAGTTAGTA
CAAACCTATAATCAGTTGTTTGAAGAGAACCCTATAAATGCAAGTGGCGTGGATGCGAAGGCTATTCTTAGCGCCCGCCTCTCTAAATCCCGACG
GCTAGAAAACCTGATCGCACAATTACCCGGAGAGAAGAAAAATGGGTTGTTCGGTAACCTTATAGCGCTCTCACTAGGCCTGACACCAAATTTT
AAGTCGAACTTCGACTTAGCTGAAGATGCCAAATTGCAGCTTAGTAAGGACACGTACGATGACGATCTCGACAATCTACTGGCACAAATTGGAG
ATCAGTATGCGGACTTATTTTTGGCTGCCAAAAACCTTAGCGATGCAATCCTCCTATCTGACATACTGAGAGTTAATACTGAGATTACCAAGGCGC
CGTTATCCGCTTCAATGATCAAAAGGTACGATGAACATCACCAAGACTTGACACTTCTCAAGGCCCTAGTCCGTCAGCAACTGCCTGAGAAATA
TAAGGAAATATTCTTTGATCAGTCGAAAAACGGGTACGCAGGTTATATTGACGGCGGAGCGAGTCAAGAGGAATTCTACAAGTTTATCAAACCC
ATATTAGAGAAGATGGATGGGACGGAAGAGTTGCTTGTAAAACTCAATCGCGAAGATCTACTGCGAAAGCAGCGGACTTTCGACAACGGTAGC
ATTCCACATCAAATCCACTTAGGCGAATTGCATGCTATACTTAGAAGGCAGGAGGATTTTTATCCGTTCCTCAAAGACAATCGTGAAAAGATTGA
GAAAATCCTAACCTTTCGCATACCTTACTATGTGGGACCCCTGGCCCGAGGGAACTCTCGGTTCGCATGGATGACAAGAAAGTCCGAAGAAACG
ATTACTCCATGGAATTTTGAGGAAGTTGTCGATAAAGGTGCGTCAGCTCAATCGTTCATCGAGAGGATGACCAACTTTGACAAGAATTTACCGA
ACGAAAAAGTATTGCCTAAGCACAGTTTACTTTACGAGTATTTCACAGTGTACAATGAACTCACGAAAGTTAAGTATGTCACTGAGGGCATGCG
TAAACCCGCCTTTCTAAGCGGAGAACAGAAGAAAGCAATAGTAGATCTGTTATTCAAGACCAACCGCAAAGTGACAGTTAAGCAATTGAAAGA
GGACTACTTTAAGAAAATTGAATGCTTCGATTCTGTCGAGATCTCCGGGGTAGAAGATCGATTTAATGCGTCACTTGGTACGTATCATGACCTCCT
AAAGATAATTAAAGATAAGGACTTCCTGGATAACGAAGAGAATGAAGATATCTTAGAAGATATAGTGTTGACTCTTACCCTCTTTGAAGATCGGG
AAATGATTGAGGAAAGACTAAAAACATACGCTCACCTGTTCGACGATAAGGTTATGAAACAGTTAAAGAGGCGTCGCTATACGGGCTGGGGAC
GATTGTCGCGGAAACTTATCAACGGGATAAGAGACAAGCAAAGTGGTAAAACTATTCTCGATTTTCTAAAGAGCGACGGCTTCGCCAATAGGAA
CTTTATGCAGCTGATCCATGATGACTCTTTAACCTTCAAAGAGGATATACAAAAGGCACAGGTTTCCGGACAAGGGGACTCATTGCACGAACAT
ATTGCGAATCTTGCTGGTTCGCCAGCCATCAAAAAGGGCATACTCCAGACAGTCAAAGTAGTGGATGAGCTAGTTAAGGTCATGGGACGTCACA
AACCGGAAAACATTGTAATCGAGATGGCACGCGAAAATCAAACGACTCAGAAGGGGCAAAAAAACAGTCGAGAGCGGATGAAGAGAATAGA
AGAGGGTATTAAAGAACTGGGCAGCCAGATCTTAAAGGAGCATCCTGTGGAAAATACCCAATTGCAGAACGAGAAACTTTACCTCTATTACCTA
CAAAATGGAAGGGACATGTATGTTGATCAGGAACTGGACATAAACCGTTTATCTGATTACGACGTCGATCACATTGTACCCCAATCCTTTTTGAA
GGACGATTCAATCGACAATAAAGTGCTTACACGCTCGGATAAGAACCGAGGGAAAAGTGACAATGTTCCAAGCGAGGAAGTCGTAAAGAAAAT
GAAGAACTATTGGCGGCAGCTCCTAAATGCGAAACTGATAACGCAAAGAAAGTTCGATAACTTAACTAAAGCTGAGAGGGGTGGCTTGTCTGA
ACTTGACAAGGCCGGATTTATTAAACGTCAGCTCGTGGAAACCCGCCAAATCACAAAGCATGTTGCACAGATACTAGATTCCCGAATGAATACG
AAATACGACGAGAACGATAAGCTGATTCGGGAAGTCAAAGTAATCACTTTAAAGTCAAAATTGGTGTCGGACTTCAGAAAGGATTTTCAATTCT
ATAAAGTTAGGGAGATAAATAACTACCACCATGCGCACGACGCTTATCTTAATGCCGTCGTAGGGACCGCACTCATTAAGAAATACCCGAAGCTA
GAAAGTGAGTTTGTGTATGGTGATTACAAAGTTTATGACGTCCGTAAGATGATCGCGAAAAGCGAACAGGAGATAGGCAAGGCTACAGCCAAA
TACTTCTTTTATTCTAACATTATGAATTTCTTTAAGACGGAAATCACTCTGGCAAACGGAGAGATACGCAAACGACCTTTAATTGAAACCAATGG
GGAGACAGGTGAAATCGTATGGGATAAGGGCCGGGACTTCGCGACGGTGAGAAAAGTTTTGTCCATGCCCCAAGTCAACATAGTAAAGAAAAC
TGAGGTGCAGACCGGAGGGTTTTCAAAGGAATCGATTCTTCCAAAAAGGAATAGTGATAAGCTCATCGCTCGTAAAAAGGACTGGGACCCGAA
AAAGTACGGTGGCTTCGATAGCCCTACAGTTGCCTATTCTGTCCTAGTAGTGGCAAAAGTTGAGAAGGGAAAATCCAAGAAACTGAAGTCAGT
CAAAGAATTATTGGGGATAACGATTATGGAGCGCTCGTCTTTTGAAAAGAACCCCATCGACTTCCTTGAGGCGAAAGGTTACAAGGAAGTAAAA
AAGGATCTCATAATTAAACTACCAAAGTATAGTCTGTTTGAGTTAGAAAATGGCCGAAAACGGATGTTGGCTAGCGCCGGAGAGCTTCAAAAGG
GGAACGAACTCGCACTACCGTCTAAATACGTGAATTTCCTGTATTTAGCGTCCCATTACGAGAAGTTGAAAGGTTCACCTGAAGATAACGAACA
GAAGCAACTTTTTGTTGAGCAGCACAAACATTATCTCGACGAAATCATAGAGCAAATTTCGGAATTCAGTAAGAGAGTCATCCTAGCTGATGCC
AATCTGGACAAAGTATTAAGCGCATACAACAAGCACAGGGATAAACCCATACGTGAGCAGGCGGAAAATATTATCCATTTGTTTACTCTTACCAA
CCTCGGCGCTCCAGCCGCATTCAAGTATTTTGACACAACGATAGATCGCAAACGATACACTTCTACCAAGGAGGTGCTAGACGCGACACTGATT
CACCAATCCATCACGGGATTATATGAAACTCGGATAGATTTGTCACAGCTTGGGGGTGAC

>PmCDA1-1xUGI (SH3 Linker-3xFLAG-PmCDA1-1xUGI)

GGTGGAGGAGGTACCGGCGGTGGAGGCTCAGCAGAATACGTACGAGCTCTGTTTGACTTCAATGGGAATGACGAGGAGGATCTCCCCTTTAAG
AAGGGCGATATTCTCCGCATCAGAGATAAGCCCGAAGAACAATGGTGGAATGCCGAGGATAGCGAAGGGAAAAGGGGCATGATTCTGGTGCCA
TATGTGGAGAAATATTCCGGTGACTACAAAGACCATGATGGGGATTACAAAGACCACGACATCGACTACAAAGACGACGACGATAAATCAGGG
ATGACAGACGCCGAGTACGTGCGCATTCATGAGAAACTGGATATTTACACCTTCAAGAAGCAGTTCTTCAACAACAAGAAATCTGTGTCACACC
GCTGCTACGTGCTGTTTGAGTTGAAGCGAAGGGGCGAAAGAAGGGCTTGCTTTTGGGGCTATGCCGTCAACAAGCCCCAAAGTGGCACCGAG
AGAGGAATACACGCTGAGATATTCAGTATCCGAAAGGTGGAAGAGTATCTTCGGGATAATCCTGGGCAGTTTACGATCAACTGGTATTCCAGCTG
GAGTCCTTGCGCTGATTGTGCCGAGAAAATTCTGGAATGGTATAATCAGGAACTTCGGGGAAACGGCACACATTGAAAATCTGGGCCTGCAA
GCTGTACTACGAGAAGAATGCCCGGAACCAGATAGGACTCTGGAATCTGAGGGACAATGGTGTAGGCCTGAACGTGATGGTTTCCGAGCACTAT
CAGTGTTGTCGGAAGATTTTCATCCAAAGCTCTCATAACCAGCTCAATGAAAACCGCTGGTTGGAGAAAACACTGAAACGTGCGGAGAAGTGG
AGATCCGAGCTGAGCATCATGATCCAGGTCAAGATTCTGCATACCACTAAGTCTCCAGCCGTTGGTCCCAAGAAGAAAAGAAAAGTCGGTACC
ATGACCAACCTTTCCGACATCATAGAGAAGGAAACAGGCAAACAGTTGGTCATCCAAGAGTCGATACTCATGCTTCCTGAAGAAGTTGAGGAG
GTCATTGGGAATAAGCCGGAAAGTGACATTCTCGTACACACTGCGTATGATGAGAGCACCGATGAGAACGTGATGCTGCTCACGTCAGATGCCC
CAGAGTACAAACCCTGGGCTCTGGTGATTCAGGACTCTAATGGAGAGAACAAGATCAAGATGCTATAA

>evoCDA1-XTEN

AGTACCGACGCCGAGTACGTGCGGATCCACGAGAAGCTGGATATCTATACATTCAAGAAGCAGTTTAGCAACAATAAGAAGTCCGTGTCTCACA
GATGCTACGTGCTGTTCGAGCTGAAGCGGAGAGGAGAGAGGCGCGCCTGTTTTTGGGGCTATGCCGTGAACAAGCCACAGTCTGGAACCGAG
AGGGGAATCCACGCAGAGATCTTCAGCATCAGGAAGGTGGAGGAGTACCTGCGCGACAACCCCGGCCAGTTTACAATCAATTGGTATAGCTCC
TGGAGCCCTTGCGCCGATTGTGCCGAGAAGATCCTGGAGTGGTACAACCAGGAGCTGAGGGGCAATGGCCACACCCTGAAGATCTGGGTGTGC
AAGCTGTACTATGAGAAGAACGCCAGGAATCAGATCGGCCTGTGGAACCTGCGCGACAATGGCGTGGGCCTGAACGTGATGGTGTCCGAGCAC
TATCAGTGCTGTCGCAAGATCTTTATCCAGTCTAGCCACAATCAGCTGAACGAGAATCGGTGGCTGGAGAAAACACTGAAGAGAGCCGAGAAG
CGGAGAAGCGAGCTGTCCATCATGTTTCAGGTGAAGATCCTGCACACCACAAAGTCTCCCGCCGTGTCTGGCGGATCTAGCGGAGGATCCTCTG
GCAGCGAGACACCAGGAACAAGCGAGTCAGCAACACCAGAGAGCAGTGGCGGCAGCAGCGGCGGCAGC

>Link-2xUGI

TCTGGTGGTTCTGGTTCGAGCGGAGGATCCGGAGGATCTGGAGGCAGCGCTTCTAGCGGGGGGAGCACTAATCTGAGCGACATCATTGAGAAG
GAGACTGGGAAACAGCTGGTCATTCAGGAGTCCATCCTGATGCTGCCTGAGGAGGTGGAGGAAGTGATCGGCAACAAGCCAGAGTCTGACAT
CCTGGTGCACACCGCCTACGACGAGTCCACAGATGAGAATGTGATGCTGCTGACCTCTGACGCCCCCGAGTATAAGCCTTGGGCCCTGGTCATC
CAGGATTCTAACGGCGAGAATAAGATCAAGATGCTGAGCGGAGGATCCGGAGGATCTGGAGGCAGCACCAACCTGTCTGACATCATCGAGAAG
GAGACAGGCAAGCAGCTGGTCATCCAGGAGAGCATCCTGATGCTGCCCGAAGAAGTCGAAGAAGTGATCGGAAACAAGCCTGAGAGCGATAT
CCTGGTCCATACCGCCTACGACGAGAGTACCGACGAAAATGTGATGCTGCTGACATCTGACGCCCCAGAGTATAAGCCCTGGGCTCTGGTCATC
CAGGATTCCAACGGAGAGAACAAAATCAAAATGCTG

>APOBEC3A-XTEN

GAGGCCAGCCCGGCTAGCGGCCCAAGGCATCTCATGGACCCGCACATCTTCACCAGCAACTTCAACAACGGCATCGGCAGGCACAAGACCTAC
TTGTGCTACGAGGTGGAGAGGCTCGACAACGGAACCTCCGTGAAGATGGACCAACACAGGGGGTTCCTCCACAACCAAGCCAAGAACCTCCT
CTGCGGCTTCTACGGCAGGCACGCCGAGTTGAGGTTCCTCGACTTGGTGCCATCCCTCCAACTCGATCCAGCCCAAATCTACCGCGTGACCTGG
TTCATCTCCTGGTCCCCATGCTTCTCCTGGGGTTGCGCCGGCGAGGTTCGGGCTTTCCTCCAAGAAAACACCCACGTCCGCCTCCGCATTTTCGC
CGCCAGGATCTATGATTACGACCCTCTCTACAAGGAGGCCCTCCAGATGCTGCGGGACGCCGGTGCTCAGGTGAGTATCATGACCTACGACGAG
TTCAAGCACTGCTGGGACACCTTCGTTGACCACCAGGGCTGCCCATTCCAACCATGGGACGGTCTGGATGAACACAGCCAAGCCTTGTCCGGC
AGGCTCCGGGCCATCCTCCAAAACCAGGGGAACTCCGGGAGCGAGACGCCAGGCACCTCCGAGTCGGCCACCCCAGAATCTCTTAAG

>ABE8e

ATGAGTGAGGTGGAGTTCTCTCACGAATACTGGATGCGACATGCTCTAACGCTAGCAAAACGAGCGAGGGATGAACGAGAGGTTCCTGTAGGA
GCAGTGTTGGTTCTGAACAACAGAGTTATTGGTGAAGGTTGGAATCGTGCTATTGGGCTTCACGACCCAACAGCCCATGCCGAAATAATGGCGC
TCAGGCAAGGAGGCTTAGTAATGCAAAACTACAGATTAATCGACGCGACCCTGTATGTCACGTTCGAGCCATGCGTTATGTGCGCGGGCGCGAT
GATTCATTCTAGAATTGGAAGGGTTGTTTTTGGGGTGAGAAATTCTAAAAGAGGTGCGGCTGGGAGTCTTATGAATGTTCTCAATTACCCTGGTA
TGAACCATCGAGTGGAAATCACGGAGGGGATTTTGGCGGACGAATGTGCAGCATTGTTATGCGATTTCTATCGTATGCCTAGGCAGGTTTTCAAC
GCTCAGAAGAAGGCGCAAAGTTCAATT

> TerL3S2P21

TCGACTCGGTACCAAATTCCAGAAAAGAGGCCTCCCGAAAGGGGGGCCTTTTTTCGTTTTGGTCCTGTT

> sfGFP-Stop-BBa_J72163 Ter

ATGCGTAAAGGCGAAGAGCTGTTCACTGGTGTCGTCCCTATTCTGGTGGAACTGGATGGTGATGTCAACGGTCATAAGTTTTCCGTGCGTGGCG
AGGGTGAAGGTGACGCAACTAATGGTAAACTGACGCTGAAGTTCATCTGTACTACTGGTAAACTGCCGGTTCCTTGGCCGACTCTGGTAACGAC
GCTGACTTATGGTGTTCAGTGCTTTGCTCGTTATCCGGACCATATGAAGCAGCATGACTTCTTCAAGTCCGCCATGCCGGAAGGCTATGTGCAGG
AACGCACGATTTCCTTTAAGGATGACGGCACGTACAAAACGCGTGCGGAAGTGAAATTTGAAGGCGATACCCTGGTAAACCGCATTGAGCTGA
AAGGCATTGACTTTAAAGAGGACGGCAATATCCTGGGCCATAAGCTGGAATACAATTTTAACAGCCACAATGTTTACATCACCGCCGATAAACAA
AAAAATGGCATTAAAGCGAATTTTAAAATTCGCCACAACGTGGAGGATGGCAGCGTGCAGCTGGCTGATCACTACCAGCAAAACACTCCAATC
GGTGATGGTCCTGTTCTGCTGCCAGACAATCACTATCTGAGCACGCAAAGCGTTCTGTCTAAAGATCCGAACGAGAAACGCGATCATATGGTTC
TGCTGGAGTTCGTAACCGCAGCGGGCATCACGCATGGTATGGATGAACTGTACAAA**TGA**CCAGGCATCAAATAAAACGAAAGGCTCAGTCGAA
AGACTGGGCCTTTCGTTTTATCTGTTGTTTGTCGGTGAACGCTCTCTACTAGAGTCACACTGGCTCACCTTCGGGTGGGCCTTTCTGCGTTTATA

References

1.Livak, K. J. & Schmittgen, T. D. Analysis of relative gene expression data using real-time quantitative PCR and the 2-ΔΔCT method. *Methods* **25**, 402–408 (2001).

2.Xing, Hui-Li, *et al.* A CRISPR/Cas9 toolkit for multiplex genome editing in plants. *BMC Plant Biol.* **14**, 1-12 (2014).

3.Lowder, L. G. *et al.* A CRISPR/Cas9 toolbox for multiplexed plant genome editing and transcriptional regulation. *Plant Physiol.* **169**, 971-985 (2015).

4.Jacobs, T. B. *et al.* Targeted genome modifications in soybean with CRISPR/Cas9. *BMC Biotechnol.* **15**, 1-10 (2015).

5.Liang, Z. *et al.* Targeted mutagenesis in Zea mays using TALENs and the CRISPR/Cas system. *J. Genet. Genomics* **41**, 63-68 (2014).

6.Lin, Q. *et al.* Prime genome editing in rice and wheat. *Nat. Biotechnol.* **38**, 582-585 (2020).

7.Shan, Q. *et al.* Targeted genome modification of crop plants using a CRISPR-Cas system. *Nat. Biotechnol.* **31**, 686-688 (2013).

8.DiCarlo, J. E. *et al.* Genome engineering in *Saccharomyces cerevisiae* using CRISPR-Cas systems. *Nucleic Acids Res.* **41**, 4336-4343 (2013).

9.Fu, Y. *et al.* High-frequency off-target mutagenesis induced by CRISPR-Cas nucleases in human cells. *Nat. Biotechnol.* **31**, 822-826 (2013).

10.Nekrasov, V. *et al.* Targeted mutagenesis in the model plant *Nicotiana benthamiana* using Cas9 RNA-guided endonuclease. *Nat. Biotechnol.* **31**, 691-693 (2013).

11.Thyme, S. B. *et al.* Internal guide RNA interactions interfere with Cas9-mediated cleavage. *Nat. Commun.* **7**, 1–7 (2016).

12.Zuker, M. Mfold web server for nucleic acid folding and hybridization prediction. *Nucleic Acids Res.* **31**, 3406-3415 (2003).

13.Pattanayak, V. *et al.* High-throughput profiling of off-target DNA cleavage reveals RNA-programmed Cas9 nuclease specificity. *Nat. Biotechnol.* **31**, 839-843 (2013).

14.Woo, J. W. *et al.* DNA-free genome editing in plants with preassembled CRISPR-Cas9 ribonucleoproteins. *Nat. Biotechnol.* **33**, 1162-1164 (2015).

15.Charrier, A. *et al.* Efficient targeted mutagenesis in apple and first time edition of pear using the CRISPR-Cas9 system. *Front. Plant Sci.* **10**, 1-12 (2019).

16. Lee, M. E., DeLoache, W. C., Cervantes, B., & Dueber, J. E. A highly characterized yeast toolkit for modular, multipart assembly. *ACS Synth. Biol.* **4**, 975-986 (2015).
17. Engler, C. *et al*. A Golden Gate modular cloning toolbox for plants. *ACS Synth. Biol.* **3**, 839–843 (2014).
18. Tsutsui, H. & Higashiyama, T. pKAMA-ITACHI vectors for highly efficient CRISPR/Cas9-mediated gene knockout in *Arabidopsis thaliana*. *Plant Cell Physiol.* **58**, 46–56 (2017).
19. Nishida, K. *et al.* Targeted nucleotide editing using hybrid prokaryotic and vertebrate adaptive immune systems. *Science* **353**, aaf8729 (2016).
20. Thuronyi, B. W. *et al.* Continuous evolution of base editors with expanded target compatibility and improved activity. *Nat. Biotechnol.* **37**, 1070-1079 (2019).
21. Zong, Y. *et al.* Efficient C-to-T base editing in plants using a fusion of nCas9 and human APOBEC3A. *Nat. Biotechnol.* **36**, 950-953 (2018).
